# Supplementary material for: A Bivalve Biomineralization Toolbox
Source: Mol Biol Evol. 2021 May 20;38(9):4043–55. doi: 10.1093/molbev/msab153 (PMC8382897; doi:10.1093/molbev/msab153)
Supplement: msab153_Supplementary_Data [file msab153_supplementary_data.pdf]

Supplemental Information S1: Analysis of differential gene expression in the asymmetrical valves of *P. maximus*.

Multidimensional scaling (MDS) plots of the digital expression levels in *P. maximus* showed a clear separation between the edge and central mantle tissues (Figure S1). This variation in expression between the edge and central mantle was consistent in both the convex and flat valves. However, the mantle edges and the central mantles of the two valves showed very little differentiation in gene expression, suggesting that the convex and flat valves have similar gene repertoire at both the edge and central mantle sections. Therefore asymmetrical morphology will not impact shell damage-repair results.

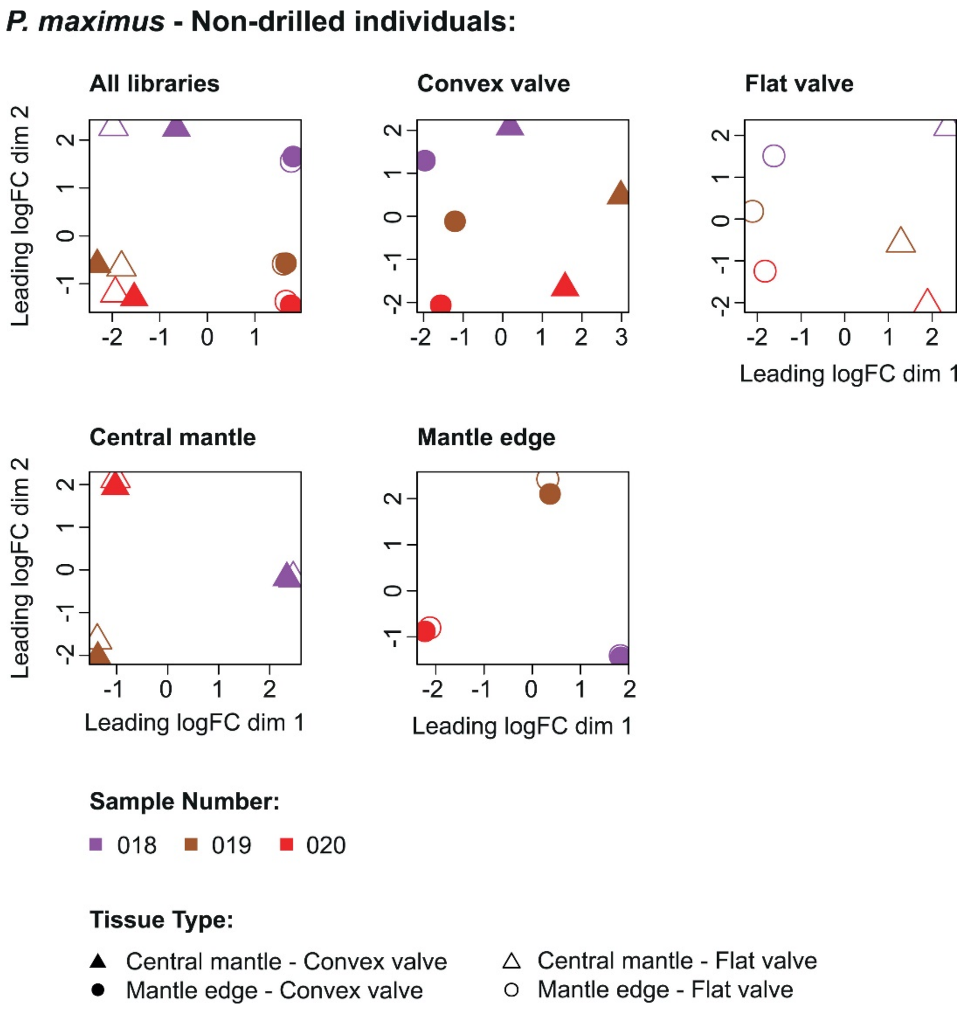

Figure S1: MDS plots of expression counts in non-drilled *P. maximus* individuals

Volcano plots of differentially expressed (DE) contigs between the four mantle sections paralleled the observations in the MDS plots. A large number of contigs were differentially expressed DE between the edge and central mantle tissues in both valves, while very few contigs were DE between the convex and at valves at both the mantle edge and central sections (Figure S2).

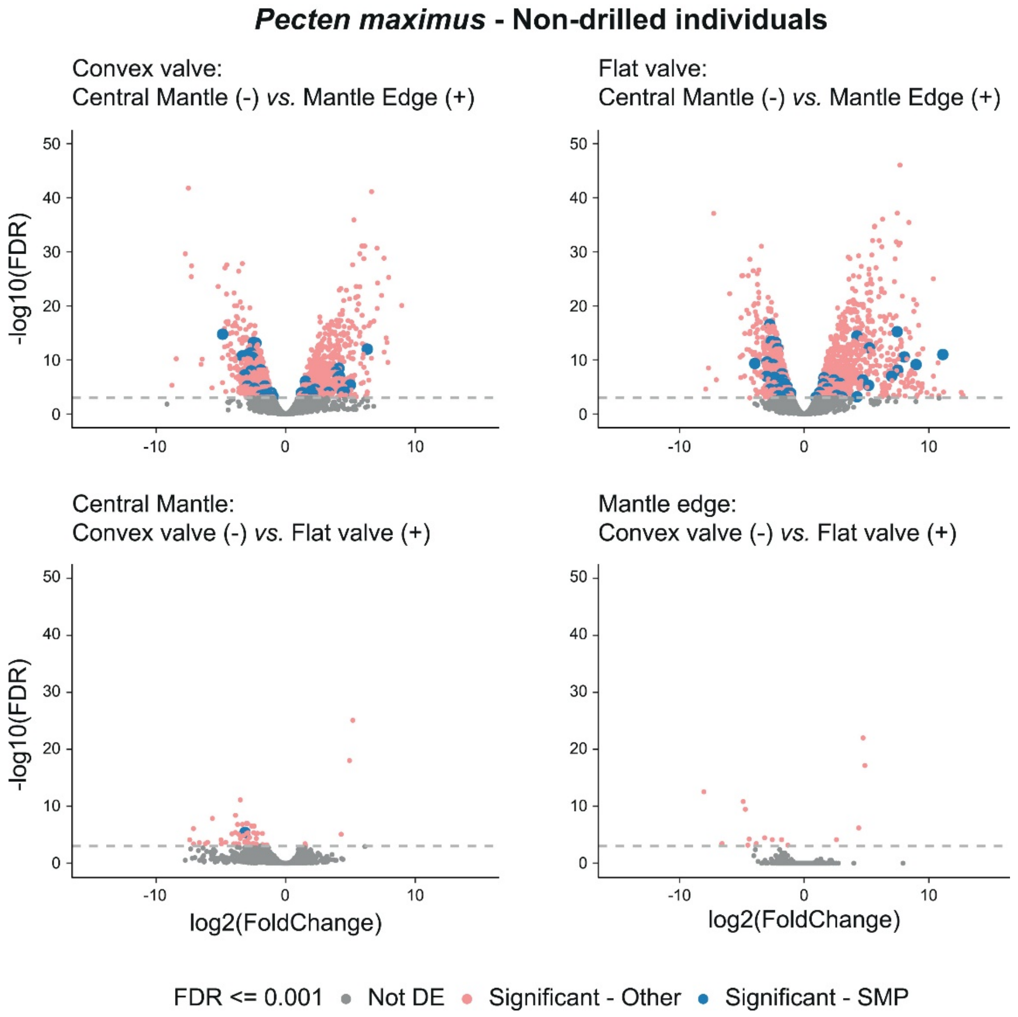

Figure S2: Volcano plots detailing differential gene expression between the four mantle tissue libraries in non-drilled *P. maximus* individuals.
Dashed lines indicate the FDR value of 0.001.

A number of contigs with strong sequence similarities to known shell matrix proteins (SMPs) were also observed to be DE between the edge and central mantle sections.

Table S1: Number of differentially expressed contigs in the different mantle tissue sections of non-drilled *P. maximus*

	P. maximus	
	**DE**	**SMP**
**Central mantle vs. Mantle edge: Convex/left valve**		
Mantle edge	1,282	20
Central mantle	1,289	22
**Central mantle vs. Mantle edge: Flat/right valve**		
Mantle edge	1,693	20
Central mantle	1,639	28
**Convex/left vs. flat/right valve: Central mantle**		
Mantle edge	52	1
Central mantle	5	0
**Convex/left vs. flat/right valve: Mantle edge**		
Mantle edge	11	0
Central mantle	4	0

In non-drilled *P. maximus* individuals, very few contigs were differentially expressed between the convex and flat valves, at either the edge or the central mantle (Table S1). However, when comparing the edge and central mantle sections in the convex and the flat valves, many differentially expressed genes were identified (1,282 + 1,289 contigs in the convex valve, and 1,693 + 1,639 contigs in the flat valve). Upon inspection, this difference was not due to simply having more contigs called as differentially expressed in the flat valve. Inspecting the normalized abundance values revealed the discrepancy to be caused by the variation in gene expression among individuals. For example, contig TRINITY DN144257 c0 g1 is identified as highly expressed in the central mantle compared to the mantle edge in the at valve, but not in the convex valve. Upon inspection of the counts, although this contig seems to be more expressed in the central mantle of the convex valve in two individuals (18,19), it was not called as differentially expressed due to individual 20 showing a different pattern in the counts (Table S2).

Table S2: Normalized abundance counts of contig TRINITY DN144257 c0 g1

	**Convex valve**		**Flat valve**	
**Individual ID**	**Central mantle**	**Mantle edge**	**Central mantle**	**Mantle edge**
**18**	600.876	321.015	1016.726	423.38
**19**	340.388	368.092	860.939	399.763
**20**	437.229	837.661	1295.855	408.231

NB: Abundance counts were normalised by TPM and TMM. Contig has sequence similarity to arginine kinase (UniProt ID: Karg_Turco (the marine gastropod: *Turbo conrnutus*, horned turban)).

Very similar enrichment of GO terms was identified in the undamaged control individuals of *P. maximus* in both the flat and the central valves in the mantle edge and the central mantle. The flat valve, acting as the control in the damaged individuals, also showed similar GO term enrichment as the undamaged individuals, although fewer terms were enriched in the mantle edge (Table S3).

Table S3: Enrichment of GO terms differentially expressed between the central and edge mantle sections in *P. maximus*. Top five Molecular Function terms.

**Non-drilled animals**<br>**Flat valve**	**Non-drilled animals**<br>**Convex valve**	**Drilled animals**<br>**Undamaged flat valve**
**Mantle edge**	**Mantle edge**	**Mantle edge**
Calcium ion binding	Calcium ion binding	Calcium ion binding
Protein binding	Protein binding	Receptor binding
Receptor binding	Receptor binding	Neurotransmitter: sodium symporter
Neurotransmitter: sodium symporter	Transporter activity	
Microtubule motor	Neurotransmitter: sodium symporter	
**Central mantle**	**Central mantle**	**Central mantle**
Structural component of ribosome	Structural component of ribosome	NADH dehydrogenase (ubiquinone)
Oxidoreductase acting on NAD(P)H	RNA binding	Oxidoreductase acting on NAD(P)H
Translation initiation factor	NADH dehydrogenase (ubiquinone)	Catalytic activity
NADH dehydrogenase (ubiquinone)	Translation initiation factor	Translation initiation factor
Proton-transporting ATP synthase	Proton-transporting ATP synthase	methyltransferase

Supplementary Information S2: MDS plots for all three species

P. maximus - Drilled individuals:

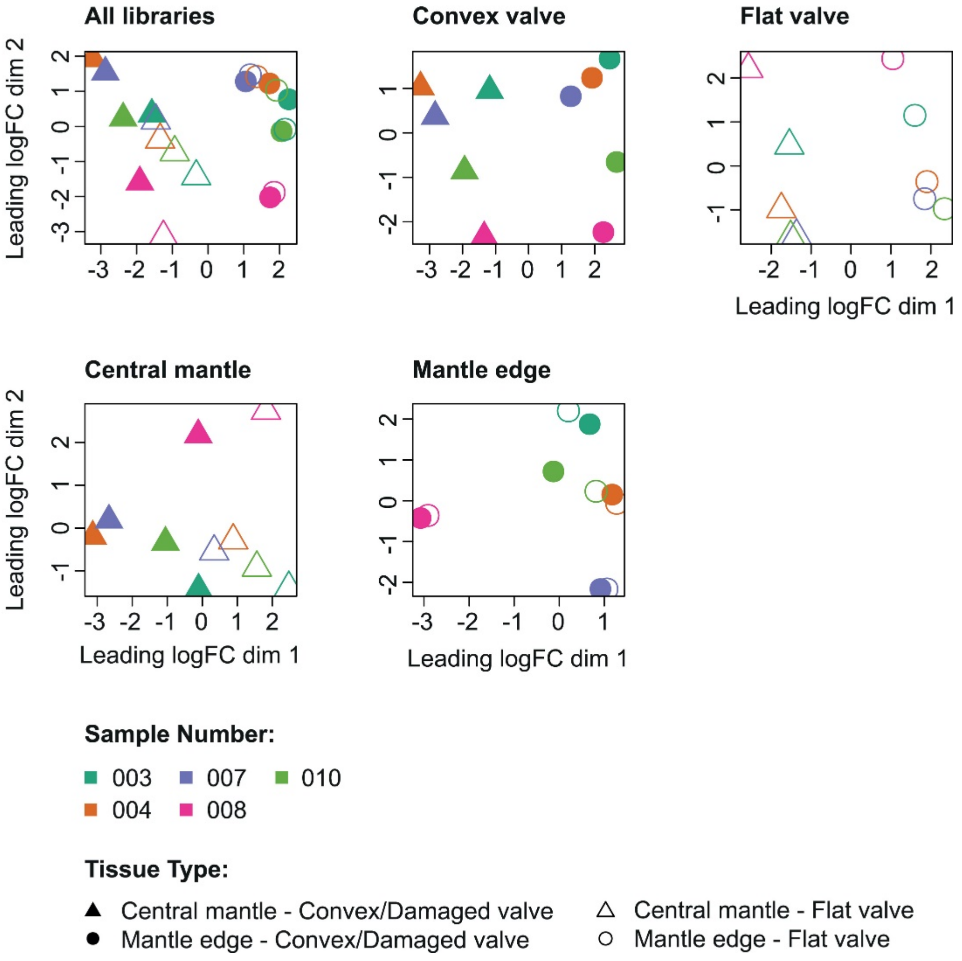

C. gigas - Drilled individuals:

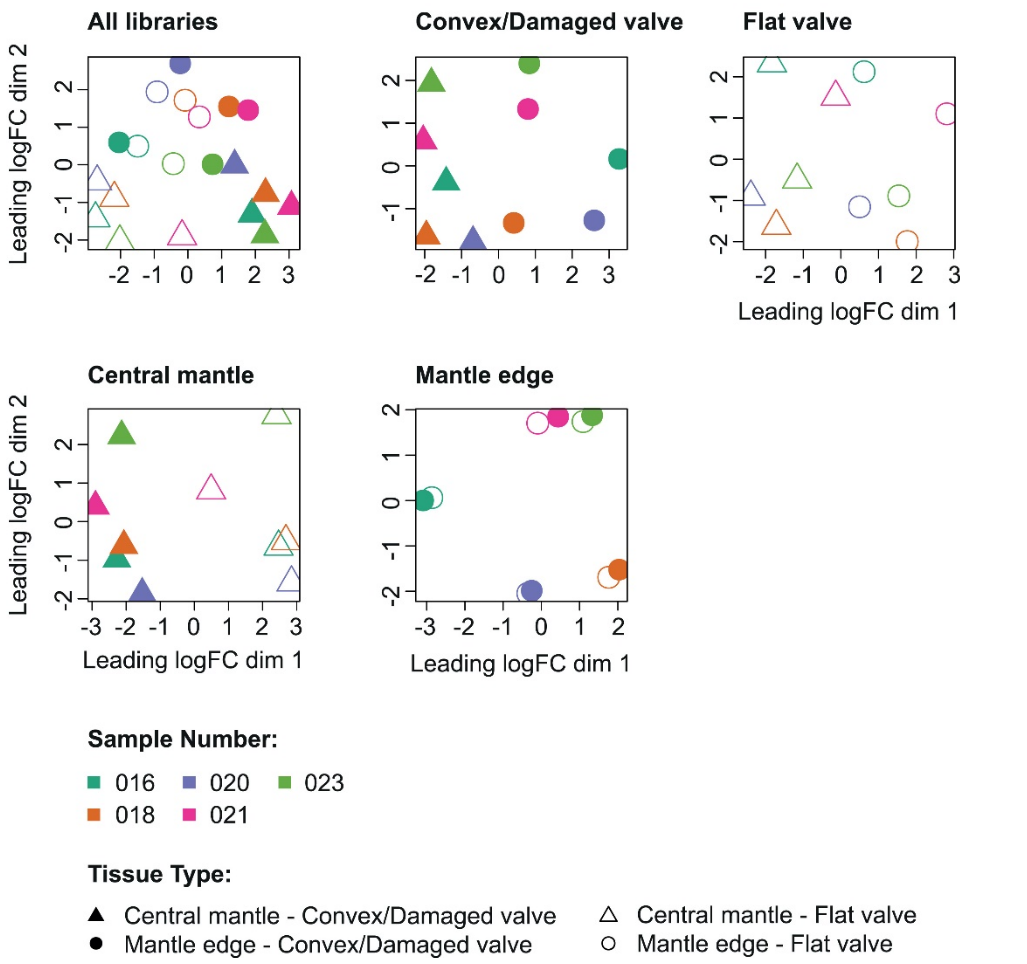

***M. edulis* - Drilled individuals:**

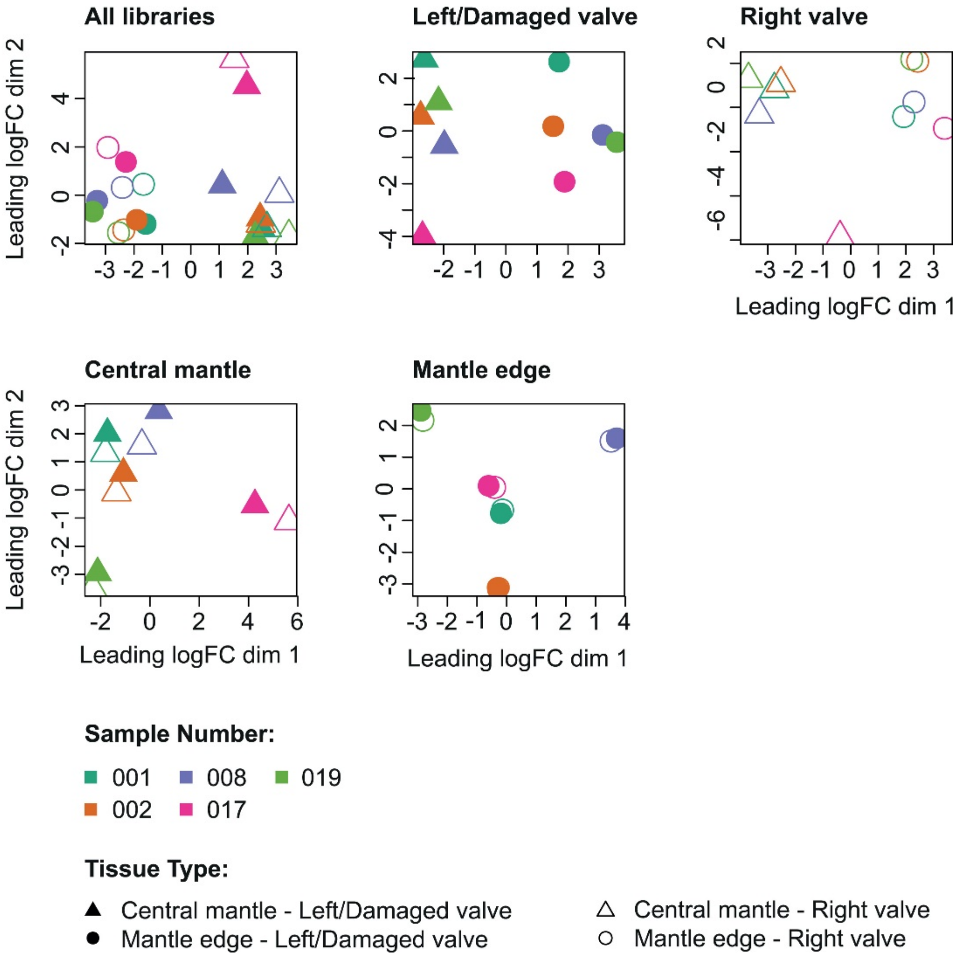

Supplementary Information S3: Volcano plots for all three species

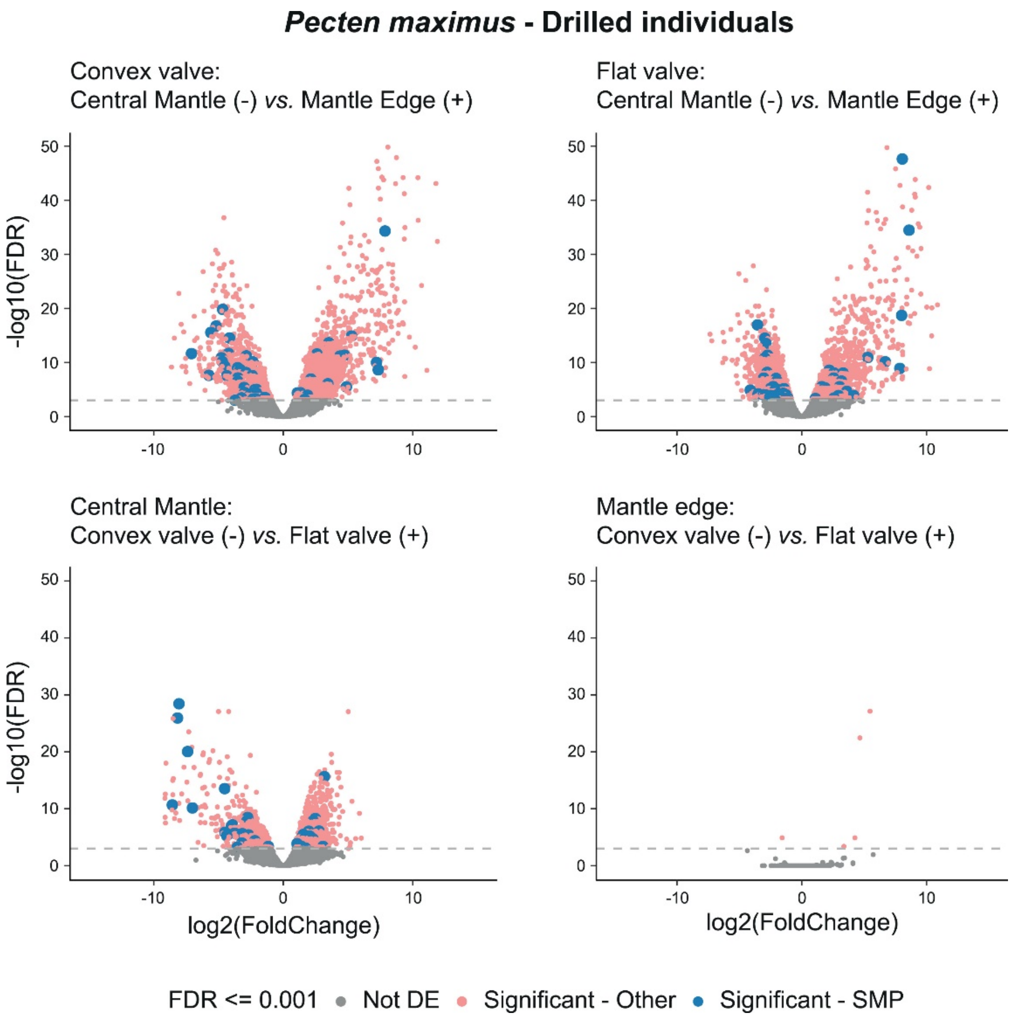

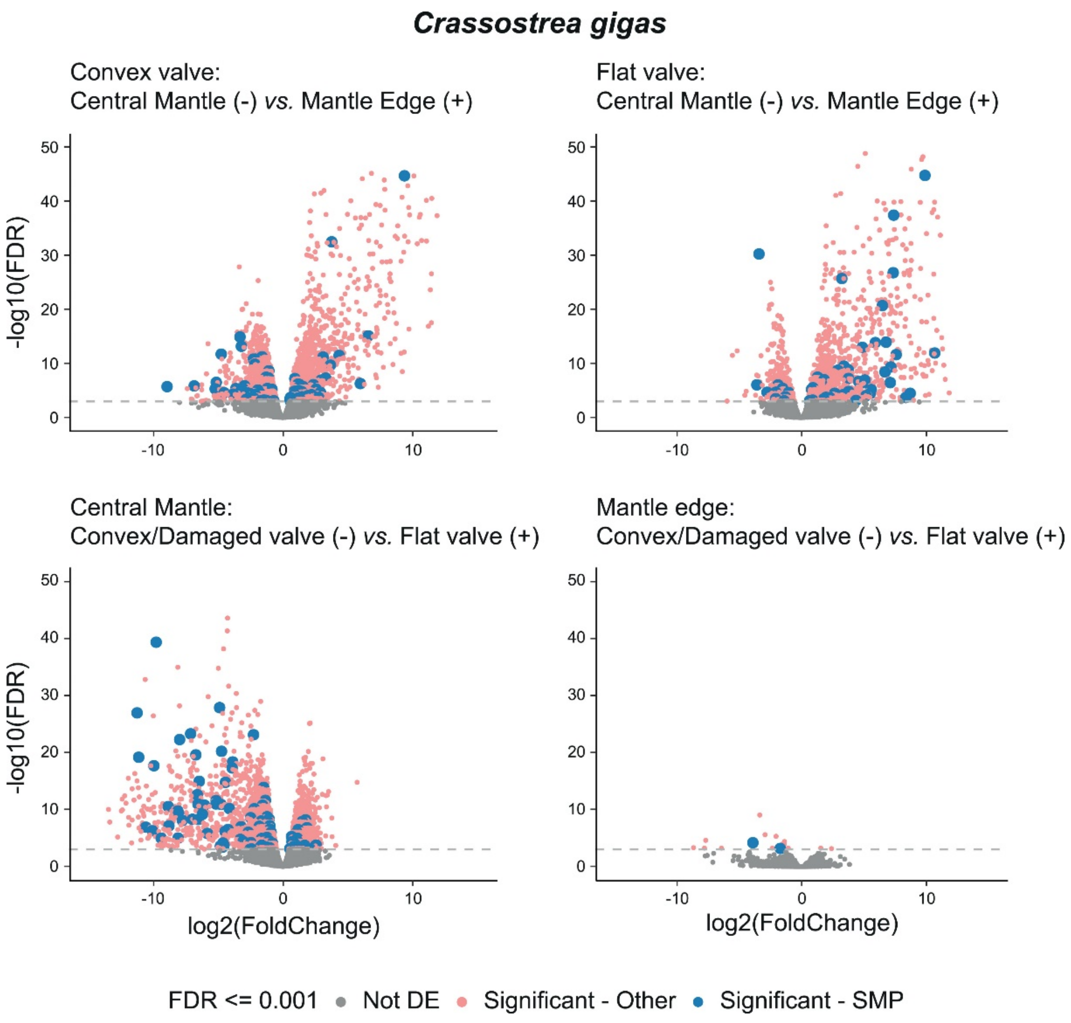
Crassostrea gigas
Convex valve:
Central Mantle (-) vs. Mantle Edge (+)
Flat valve:
Central Mantle (-) vs. Mantle Edge (+)
Central Mantle:
Convex/Damaged valve (-) vs. Flat valve (+)
Mantle edge:
Convex/Damaged valve (-) vs. Flat valve (+)
-log10(FDR)
log2(FoldChange)
50
40
30
20
10
0
-10
0
10
FDR <= 0.001
Not DE
Significant - Other
Significant - SMP

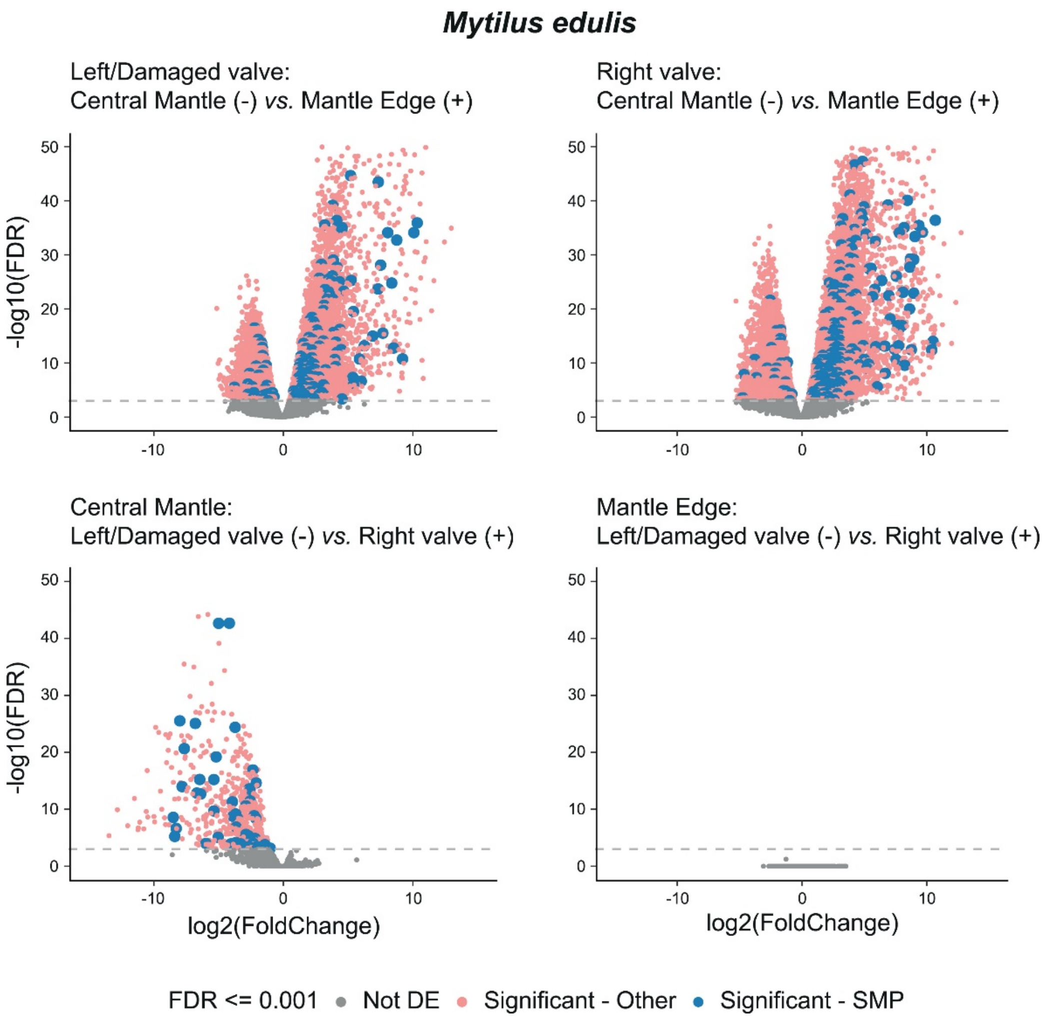

Mytilus edulis
Left/Damaged valve:
Central Mantle (-) vs. Mantle Edge (+)
Right valve:
Central Mantle (-) vs. Mantle Edge (+)
Central Mantle:
Left/Damaged valve (-) vs. Right valve (+)
Mantle Edge:
Left/Damaged valve (-) vs. Right valve (+)
-log10(FDR)
log2(FoldChange)
50
40
30
20
10
0
-10
0
10
FDR <= 0.001
Not DE
Significant - Other
Significant - SMP

Supplementary Information S4: Enrichment of GO terms in contigs differentially expressed between the damaged and undamaged central mantle tissue sections. Top five Molecular Function (MF) terms.

P. maximus	***C. gigas***	***M. edulis***
**Damaged/convex valve**	**Damaged/convex valve**	**Damaged/convex valve**
None	Unfolded protein binding	Peptidase inhibitor activity
	Translation initiation factor activity	Chitin binding
	Endopeptidase activity	Serine-type endopeptidase activity
	Threonine-type endopeptidase activity	Metalloendopeptidase inhibitor
	Aminoacyl-tRNA ligase activity	
**Undamaged/flat valve**	**Undamaged/flat valve**	**Undamaged/flat valve**
NADH dehydrogenase (ubiquinone)	Ubiquitin-protein transferase activity	None
Oxidoreductase acting on NAD(P)H	Protein tyrosine phosphatase activity	
Proton-transporting ATP synthase	Deoxyribonuclease II activity	
Rotational mechanism	Tumour necrosis factor receptor binding	
